# Brassinosteroids is involved in methane-induced adventitious root formation via inducing cell wall relaxation in marigold

**DOI:** 10.1186/s12870-022-04014-9

**Published:** 2023-01-02

**Authors:** Yihua Li, Jun Hua, Xuemei Hou, Nana Qi, Changxia Li, Chunlei Wang, Yandong Yao, Dengjing Huang, Hongsheng Zhang, Weibiao Liao

**Affiliations:** 1 College of Horticulture, Gansu Agricultural University, 1 Yingmen Village, Anning District, Lanzhou, 730070 China; 2 College of Agriculture and Ecological Engineering, Hexi University, No.846 Beihuan Road, Zhangye, 734000 Gansu China; 3 Cash-Crops Technology Extension Centre of Zhangye City, No.675 Nanhuan Road, Zhangye, 734000 Gansu China; 4 College of Agriculture, Guangxi University, No.100 East University Road, Nanning, 530004 China

**Keywords:** Methane-rich water, 2,4-epibrassinolide, Adventitious root formation, Cell wall relaxation

## Abstract

**Background:**

Methane (CH_4_) and brassinosteroids (BRs) are important signaling molecules involved in a variety of biological processes in plants.

**Results:**

Here, marigold (*Tagetes erecta* L. ‘Marvel’) was used to investigate the role and relationship between CH_4_ and BRs during adventitious root (AR) formation. The results showed a dose-dependent effect of CH_4_ and BRs on rooting, with the greatest biological effects of methane-rich water (MRW, CH_4_ donor) and 2,4-epibrassinolide (EBL) at 20% and 1 μmol L^− 1^, respectively. The positive effect of MRW on AR formation was blocked by brassinoazole (Brz, a synthetic inhibitor of EBL), indicating that BRs might be involved in MRW-regulated AR formation. MRW promoted EBL accumulation during rooting by up-regulating the content of campestanol (CN), cathasterone (CT), and castasterone (CS) and the activity of Steroid 5α-reductase (DET2), 22α-hydroxylase (DWF4), and BR-6-oxidase (BR6ox), indicating that CH_4_ could induce endogenous brassinolide (BR) production during rooting. Further results showed that MRW and EBL significantly down-regulated the content of cellulose, hemicellulose and lignin during rooting and significantly up-regulated the hydrolase activity, i.e. cmcase, xylanase and laccase. In addition, MRW and EBL also significantly promoted the activity of two major cell wall relaxing factors, xyloglucan endotransglucosylase/hydrolase (XTH) and peroxidase, which in turn promoted AR formation. While, Brz inhibited the role of MRW on these substances.

**Conclusions:**

BR might be involved in CH_4_-promoted AR formation by increasing cell wall relaxation.

## Background

Brassinosteroids (BRs) are polyhydroxylated sterol derivatives that are classified as a new class of plant hormones with a structure very similar to that of animal and insect steroid hormones [1]. It was found that the BR-specific biosynthetic precursor campesterol (CR) is converted to brassinolide (BR) via two parallel pathways, named the campestanol (CN)-dependent pathway and the CN-independent pathway [2]. Up to now, more than 70 BR-related phytosteroids have been recognized from plants, among which brassinolide, 2,4-epibrassinolide, and 2,8-homobrassinolide have the highest biological activity [3]. BRs are widely distributed in plants and can regulate plant growth and development [4]. Previous studies have shown that BR is involved in various plant growth and development processes, including promoting cell elongation and growth, cell division, photomorphogenesis, seed germination, vascular bundle differentiation, stomatal development, and root growth [5], lateral root and AR formation [6], and fruit maturation [7]. In addition, BR regulates plants against abiotic stresses including droughty, high-temperature, low-temperature, salinity, osmotic stress and metal stress [8].

Methane (CH_4_), a significant greenhouse gas after carbon dioxide, is a volatile, colorless, odorless, and ubiquitous gaseous molecule. Normally, this gas is considered to be a physiologic inert gas [9]. However, previous reviews reported the biological effect of CH_4_, especially the protective effects of anti-inflammatory, antioxidant, and antiapoptosis [10]. It is proposed that CH_4_ may be a new functional gas in medical applications [11]. As a signal molecule, CH_4_ may be produced by an aerobic, non-microbial pathway in plants [9]. CH_4_ may be involved in plant growth and development, such as seed germination, seedling growth, adventitious rooting, lateral rooting, and postharvest freshness [12]. Zhu et al. demonstrated that CH_4_ had interaction with reactive oxygen species (ROS) [13]. Further results showed that CH_4_ may interact with other signaling molecules in the induction of ARs formation, including hydrogen sulfide (H_2_S), nitric oxide (NO), auxin, carbon monoxide (CO), hydrogen peroxide (H_2_O_2_), calcium (Ca^2+^), and glutathione (GSH) [9].

The formation of plant cell wall involves the processes of cell wall relaxation and remodeling, synthesis, assembly and deposition of cell wall components [14]. The plant cell wall enlargement is an important step of plant growth, development and morphogenesis. The key physical event required for cell enlargement is wall stress relaxation, known as cell wall relaxation [15]. Cell wall relaxation is the biophysical aspect of so-called ‘cell wall loosening’. Cell wall loosening implies a rearrangement of the load-bearing bonds, which has to occur to relax (reduce) wall stress and to enable the polymer slippage as water is absorbed into the cell and the wall swells. Wall loosening agents found so far are expansin, xyloglucan endotransglucosylase/hydrolase (XTH), cellulase, and peroxidase (POD). Using *Arabidopsis* BR mutants, Xie et al. demonstrated that BR regulated cellulose synthase by combining with the upstream components of cellulose synthase [16]. BR could promote the addition of new xyloglucan to the cell wall, resulting in cellular relaxation and cell elongation [17].

Adventitious roots (ARs) are formed from stems, hypocotyls, buds, leaves, and other tissues, and belong to post-embryonic development. The formation of ARs is one of the important ways for plant vegetative reproduction. AR formation is a complex organogenesis process and is jointly regulated by external environmental conditions and endogenous factors, including light, temperature, moisture, mineral nutrients, plant hormones, carbohydrates, etc. [18]. Previous studies have shown that strigolactone, melatonin, and brassinolide are involved in AR formation [19]. BRs plays an important role in plant growth and development in a concentration-dependent manner. Low concentrations of BL facilitated an increase in the number and length of ARs, whereas high concentrations of BL resulted in trichome roots [20]. *Arabidopsis* seedlings treated with 24-epibrassinolide (BL) exhibited increased lateral roots (LR) density and inhibition of primary root (PR) elongation [21]. Maurini and Shibaoka showed for the first time that brassinolide alone or in combination with auxin facilitated the maintenance of microtubules oriented at the cellular level in the small bean cotyledons epidermis, and accordingly promoted plant growth [22]. Auxin and brassinosteroids could affect the expression of genes encoding enzymes that loosen the cell wall [23]. BRs also could regulate auxin transport by affecting the sorting and accumulation of PIN auxin efflux carriers [24, 25]. Exogenous promoted AR formation by inducing endogenous nitric oxide (NO) production in cucumber (*Cucumis sativus* L), and nitrate reductase (NR) and NO synthase (NOS-like) enzyme activity was involved in BR signaling [19].

Marigold (*Tagetes erecta* L.) is known as a commercially important ornamental plant in many parts of the world. Its petals are often used to extract lutein and the flowers are used in medicinal processing, pharmaceutical and food production. In addition, marigold is also used in agricultural production as a very valuable intercrop [26]. As mentioned above, both CH_4_ and BRs could promote AR development. However, it is unclear whether the crosstalk between CH_4_ and BRs is involved in the formation of ARs. Therefore, in this study, marigold was used to explore the effect of methane on the synthesis pathway of endogenous brassinolide in marigold explants. The effects and relationship of methane and BRs during AR formation were determined. Thus, this study may provide theoretical support for regulating the formation of ARs in plants.

## Results

### Effects of exogenous EBL and MRW on adventitious root development

**Fig. 1:**
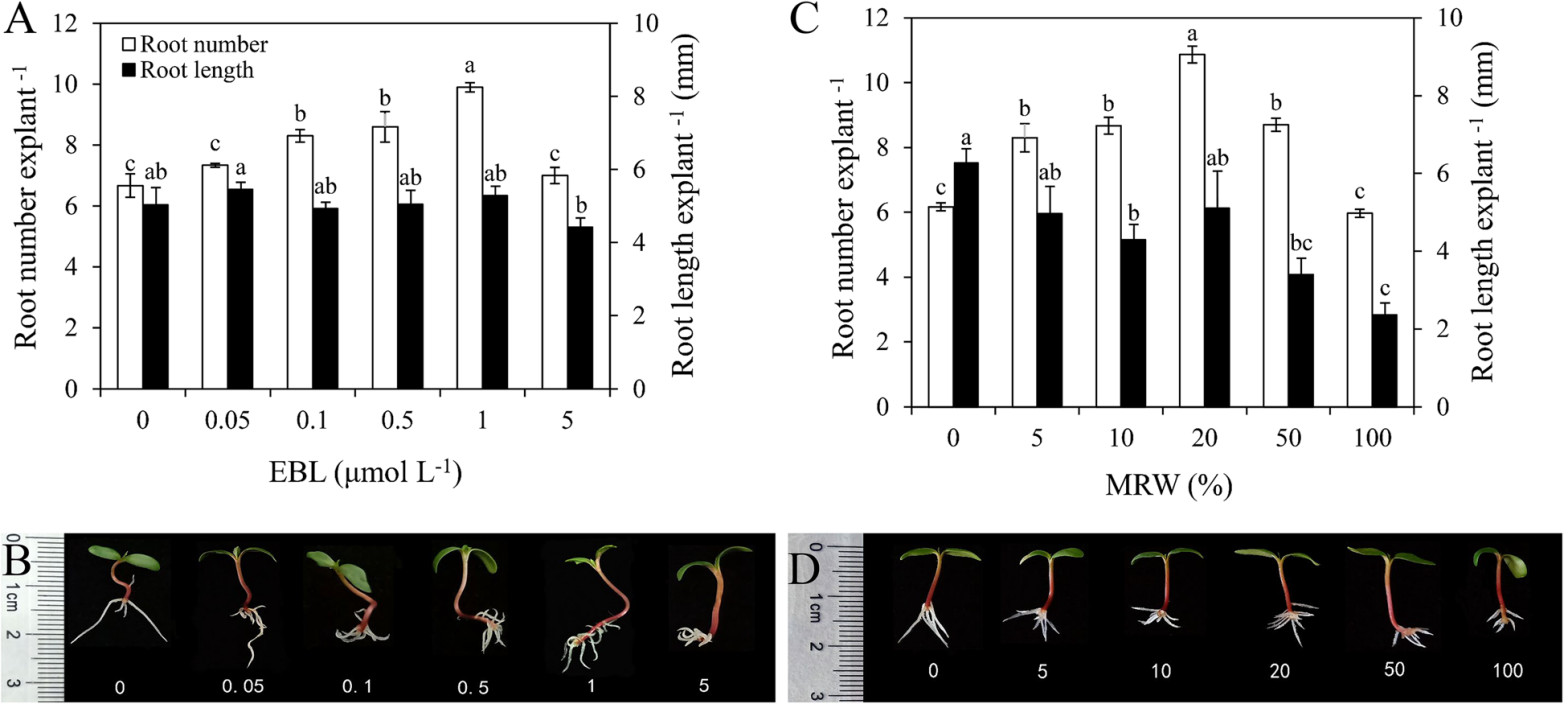
Effect of exogenous 2,4-epibrassinolide (EBL) and methane-rich water (MRW) on AR development in marigold explants. The primary roots of 5-day-old seedlings were removed, and then each of 10 marigold explants was incubated with different concentrations of EBL (0, 0.05, 0.1, 0.5, 1, 5 μmol L^− 1^) and MRW (0, 5, 10, 20, 50, 100%) for 5 days. Values (mean ± SE) of root number and root length (**A**, **C**) of marigold explants are the means of three independent experiments (*n* = 30). Bars with different lowercase letters are significantly different (Duncan’s multiple range test; *p* < 0.05). Photographs (**B**, **D**) were taken after 5 days of treatments

To elucidate the effects of EBL and MRW on adventitious rooting, a dose-response experiment with EBL and MRW was completed (Fig. 1). As compared with the control, EBL significantly increased root number at 0.1, 0.5 and 1 μmol L^− 1^, and the maximum root number was observed with the 1 μmol L^− 1^ EBL (Fig. 1A and B). However, different concentrations of EBL did not affect root length (Fig. 1A). Therefore, 1 μmol L^− 1^ EBL was used for further study.

Treatment with 100% MRW had no significant effects on root number when compared with the control, but treatments with 5, 10, 20 and 50% MRW significantly increased root number, and the maximum root number was achieved with 20% MRW treatment (Fig. 1C). Meanwhile, although treatments with 10, 50 and 100% MRW significantly decreased root length, there was no significant difference in root length among the control, 5 and 20% MRW (Fig. 1D). Thus, 20% MRW was used for further study.

### Involvement of EBL in MRW-regulated adventitious root development

**Fig. 2:**
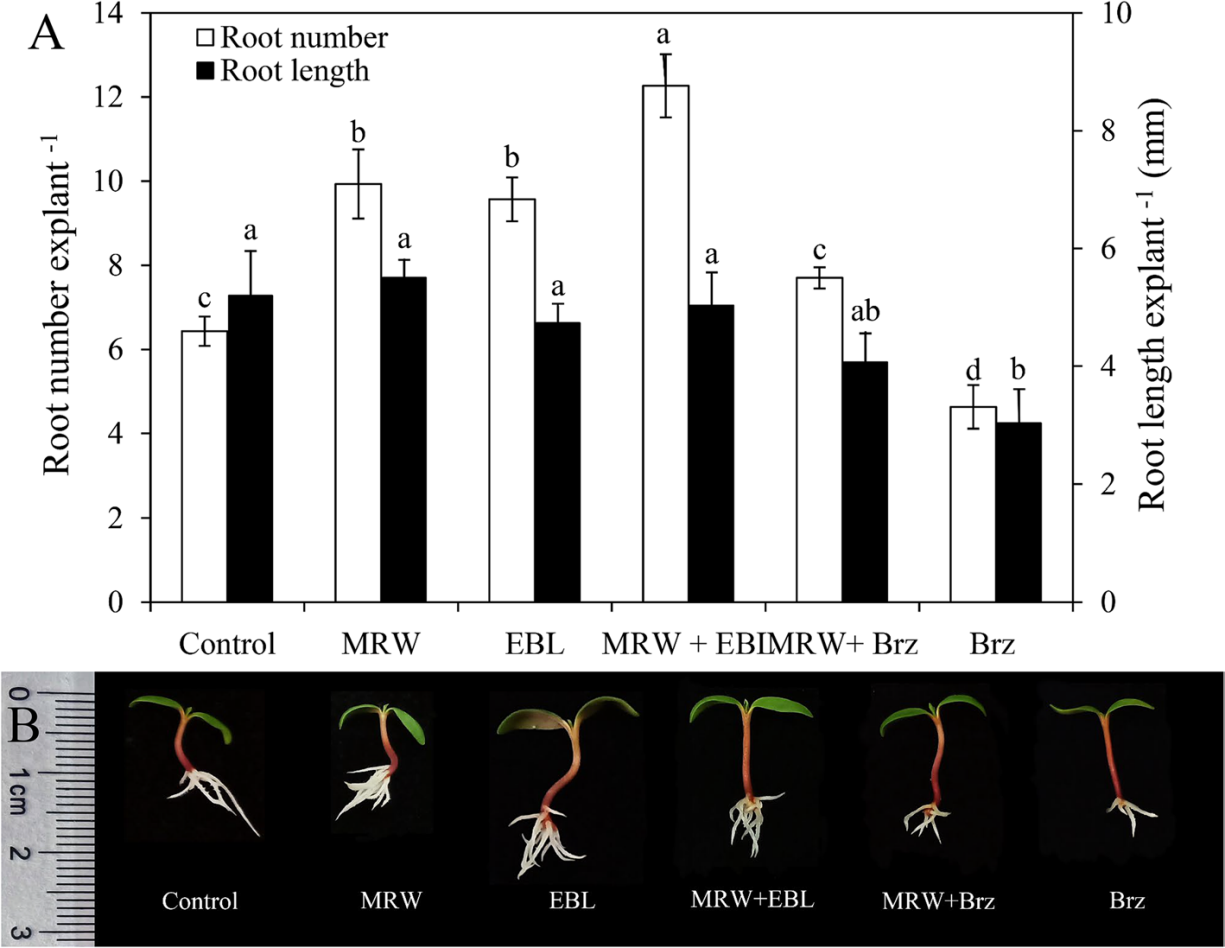
Effect of exogenous 2,4-epibrassinolide (EBL) and methane-rich water (MRW) on AR development in marigold explants. The primary roots of 5-day-old seedlings were removed, and then each of 10 marigold explants was incubated with 1 μmol L^− 1^ EBL, 20% MRW, 0.5 μmol L^− 1^ Brz (brassinazole, a synthetic inhibitor of EBL) alone or together for 5 days, as indicated. Values (mean ± SE) of root number and root length (**A**) of marigold explants are the means of three independent experiments (*n* = 30). Bars with different lowercase letters are significantly different (Duncan’s multiple range test; *p* < 0.05). Photographs (**B**) were taken after 5 days of treatments

To further investigate the relationship between EBL and methane during rooting, marigold explants were treated with 0.5 μmol L^− 1^ Brz (a synthetic inhibitor of EBL), 20% MRW, 1 μmol L^− 1^ EBL alone or together (Fig. 2). The application of MRW or EBL alone significantly increased AR number, and co-treatment of them resulted in higher root number in comparison with either MRW or EBL alone (Fig. 2A). There was no significant difference in root length among these treatments (Fig. 2A). Meanwhile, compared with the control, the application of Brz significantly reduced the number and the length of roots. The root number in MRW + Brz treatment was significant lower than that in MRW treatment (Fig. 2A, B). These results indicate that EBL might be involved in MRW-induced adventitious rooting.

### Effects of MRW on EBL biosynthesis during adventitious rooting

**Fig. 3:**
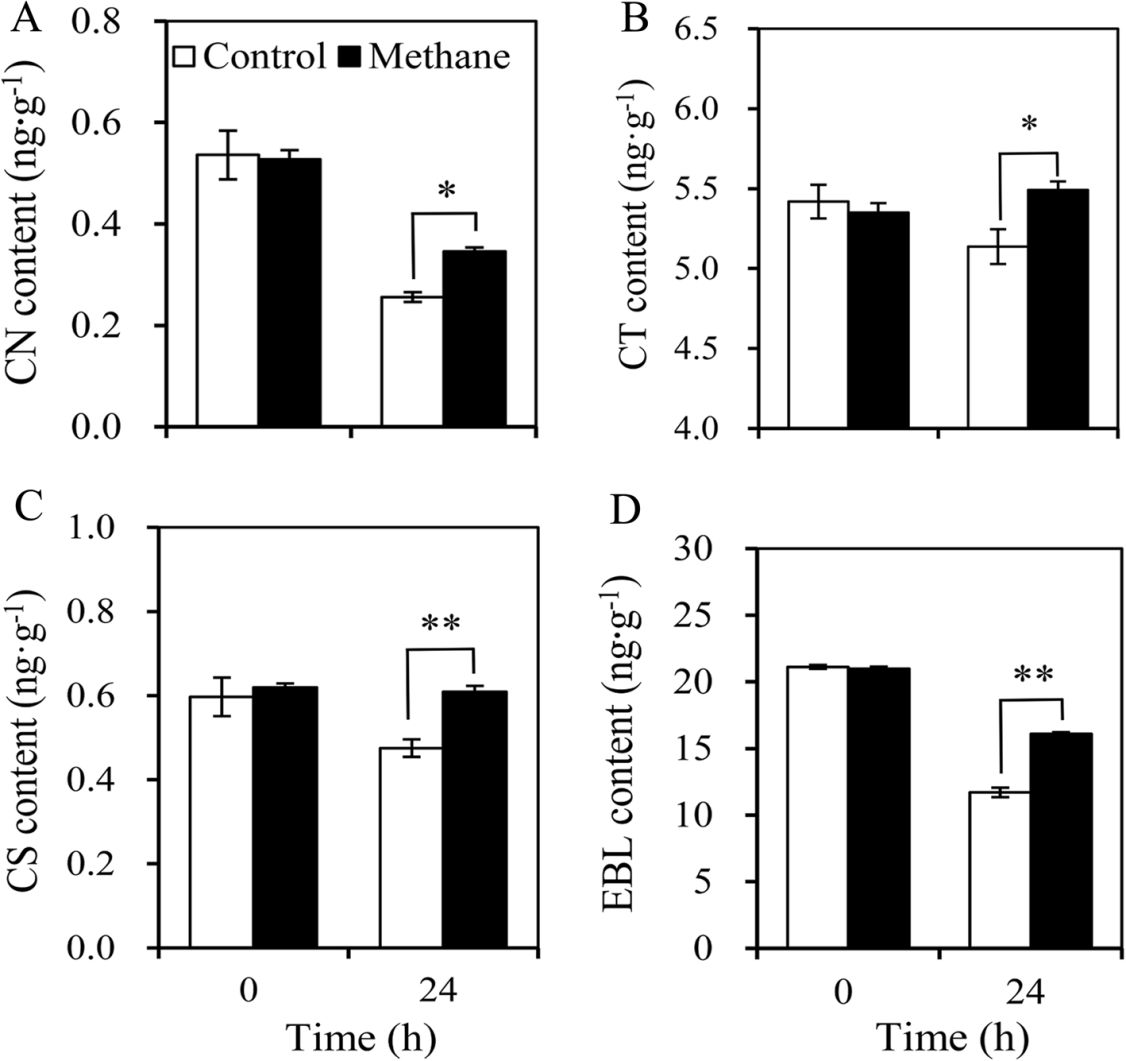
Effect of exogenous methane-rich water (MRW) on the content of endogenous campestanol (CN), cathasterone (CT), castasterone (CS), and 2,4-epibrassinolide (EBL) during AR development in marigold explants. The primary roots of 5-day-old seedlings were removed, and then the marigold explants were incubated with 20% MRW for 24 h. The content of CN (**A**), CT (**B**), CS (**C**), and EBL (**D**) was measured at 0 and 24 h after treatment. Values (mean ± SE) are the means of three independent experiments (*n* = 30). * and ** indicate significant difference and highly significant difference (independent T-test, *p* < 0.05 or *p* < 0.01), respectively

To understand the effect of CH_4_ on endogenous BR during rooting, the content of EBLand related synthetic precursor substances in marigold explants treated with MRW was determined. The content of CN, CT, CS, and EBL in MRW treatment at 24 h was significantly higher than that in the control (Fig. 3). Thus, MRW may effectively increase CN, CT, CS, and EBL levels during adventitious rooting.

**Fig. 4:**
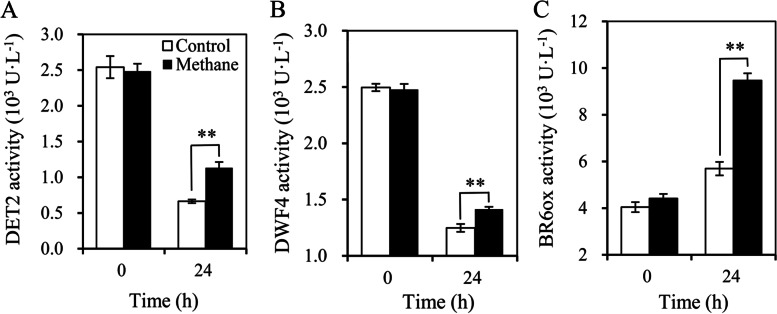
Effect of exogenous methane-rich water (MRW) on the enzymatic activities of steroid 5α-reductase (DET2), 22α-hydroxylase (DWF4) and BR-6-oxidase (BR6ox) during AR development in marigold explants. The primary roots of 5-day-old seedlings were removed, and then the marigold explants were incubated with 20% MRW for 24 h. The activities of DET2 (**A**), DWF4 (**B**), and BR6ox (**C**) were measured at 0 and 24 h after treatment. Values (mean ± SE) are the means of three independent experiments (*n* = 30). ** indicates highly significant differences (independent T-test, *p* < 0.01)

The activity of DET2, DWF4, and BR6ox in MRW treatment at 24 h was significantly higher than that in the control (Fig. 4), indicating that MRW may effectively enhance the activities of EBL biosynthesis-related enzymes during adventitious rooting.

### Effects of MRW and EBL on the contents of Cell Wall components during adventitious rooting

**Fig. 5:**
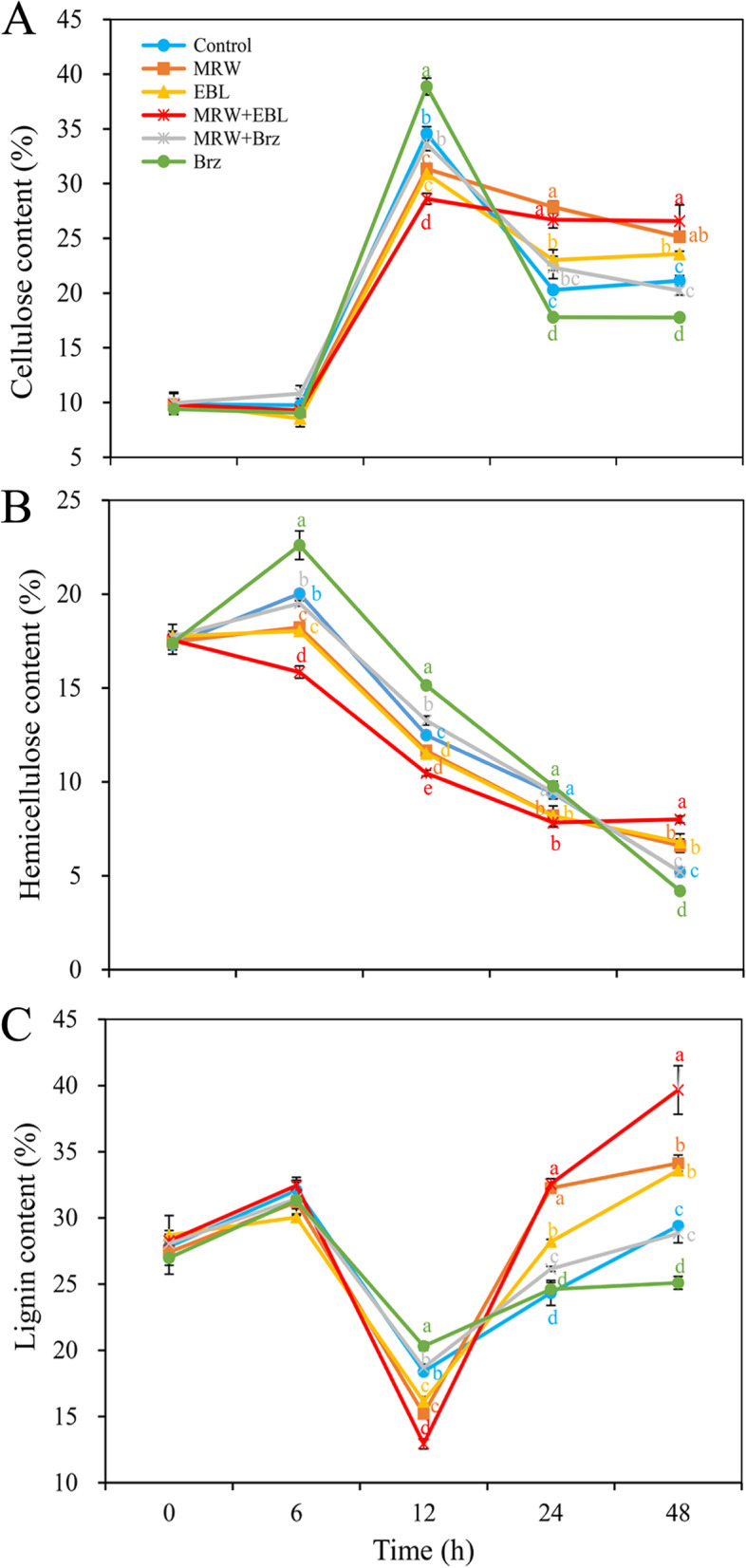
Effect of exogenous 2,4-epibrassinolide (EBL), methane-rich water (MRW), and brassinazole (Brz) on cellulose hemicellulose and lignin content during AR development in marigold explants. The primary roots of 5-day-old seedlings were removed, and then the marigold explants were incubated with 1 μmol L^− 1^ EBL, 20% MRW, 0.5 μmol L^− 1^ Brz (a synthetic inhibitor of EBL) alone or together for 48 h, as indicated. The contents of cellulose (**A**), hemicellulose (**B**), and lignin (**C**) were measured at 0, 6, 12, 24, and 48 h after treatment. Values (mean ± SE) are the means of three independent experiments (*n* = 500). Bars with different lowercase letters are significantly different (Duncan’s multiple range test; *p* < 0.05)

To explore whether BR has an effect on the relaxation of cell walls during AR formation, the contents of cellulose, hemicellulose and lignin in marigold explants were evaluated. As shown in Fig. 5A, the cellulose content of all treatments increased sharply from 6 to 12 h, and then decreased steeply from 12 to 24 h. The cellulose content in MRW, EBL and MRW + EBL treatments significantly lower than that in the control at 12 h. At 12 h, Brz treatment obtained the highest cellulose content, but MRW + EBL treatment had the lowest content (Fig. 5A).

As shown in Fig. 5B, the hemicellulose content in all treatments except MRW + EBL treatment increased from 0 to 6 h, and then decreased gradually from 6 to 24 h. From 6 to 24 h, compared with the control, MRW + EBL, MRW, and EBL treatments resulted in lower content of hemicellulose. MRW + EBL treatment showed the minimum level of hemicellulose among all treatments. However, From 6 to 12 h, Brz treatment obtained the maximum hemicellulose content among all treatments. Similarly, at 48 h, the hemicellulose content in Brz treatment was lower than that in other treatments, while the MRW + EBL treatment had the highest hemicellulose content among all treatments (Fig. 5B).

In all treatments, the lignin content decreased from 6 to 12 h, and then increased from 12 to 48 h (Fig. 5C). The lignin content in all treatments was the lowest at 12 h. At 12 h, compared with the control, MRW, EBL or MRW + EBL treatments decreased the lignin content. Meanwhile, MRW + EBL treatment resulted in lower lignin content than either MRW or EBL alone. However, compared with the control, Brz treatment significantly increased lignin content at 12 h (Fig. 5C). At 48 h, compared with the control, the lignin content in MRW, EBL, and MRW + EBL treatments was increased by 16.1, 14.3, and 34.9%, respectively. However, Brz treatment decreased lignin content by 14.6%.

### Effects of MRW and EBL on the activities of hydrolase during adventitious rooting

**Fig. 6:**
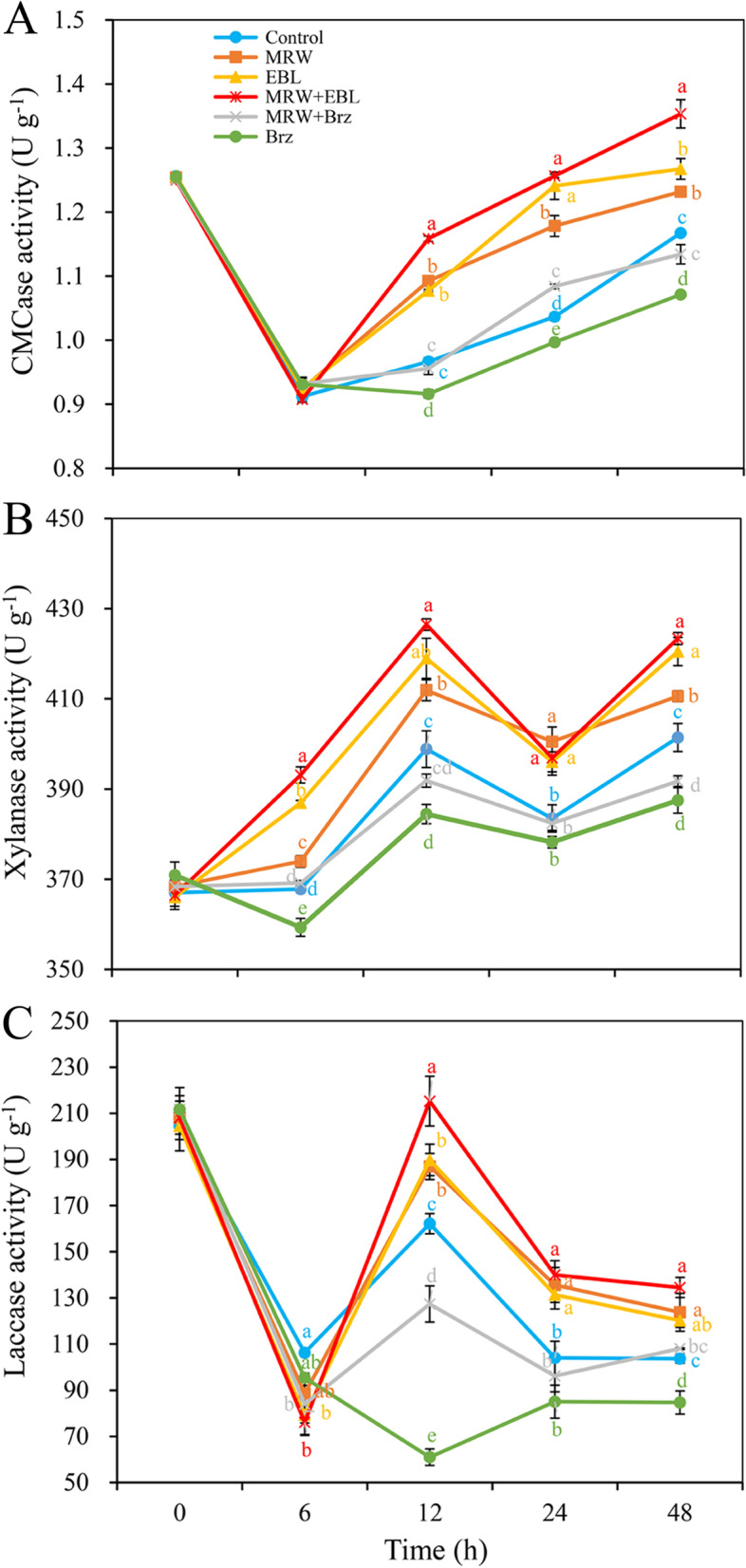
Effect of exogenous 2,4-epibrassinolide (EBL), methane-enriched water (MRW) and brassinazole (Brz) on the CMCase, xylanase and laccase activity during AR development in marigold explants. The primary roots of 5-day-old seedlings were removed, and then the marigold explants were incubated with 1 μmol L^− 1^ EBL, 20% MRW, and 0.5 μmol L^− 1^ Brz (a synthetic inhibitor of EBL) alone or together for 48 h, as indicated. The activities of carboxymethyl cellulose (CMCase, **A**), xylanase (**B**), laccase (**C**) were measured at 0, 6, 12, 24 and 48 h after treatment. Values (mean ± SE) are the means of three independent experiments (*n* = 200). Bars with different lowercase letters are significantly different (Duncan’s multiple range test; *p* < 0.05)

The CMCase activity decreased sharply in the early stage of rooting (from 0 to 6 h), and then it increased (from 12 to 48 h; Fig. 6A). Between 12 and 48 h, the CMCase activity was higher in MRW + EBL-, MRW-, or EBL-treated explants than in the control explants. Among all treatments, the maximum CMCase activity was obtained in MRW + EBL treatment from 12 to 48 h, particularly at 12 and 48 h. Meanwhile, the CMCase activity in Brz treatment was significantly lower than that in the control from 12 to 48 h (Fig. 6A).

The xylanase activity in all treatments showed an increase trend from 0 to 12 h, and an obviously decrease trend from 12 to 24 h, and a gradual increase trend from 24 to 48 h (Fig. 6B). Notably, MRW + EBL co-treatment had the highest xylanase activity among all treatments at 6, 12, and 48 h, which was significantly higher than MRW or EBL alone treatment. The xylanase activity in MRW or EBL treatment was higher than that in the control. While, Brz treatment resulted in the lowest xylanase activity in all treatments (Fig. 6B).

As shown in Fig. 6C, the laccase activity in the control, MRW, EBL, and MRW + EBL treatments decreased sharply from 0 to 6 h, and increased sharply from 6 to 12 h, then decreased again from 12 to 24 h, and steadily decreased from 24 to 48 h. MRW or EBL treatment significantly enhanced laccase activity compared with the control between 12 and 48 h. MRW and EBL co-treatment had higher laccase activity than MRW or EBL alone treatment between 12 and 48 h, at the same time, Brz treatment significantly reduced the laccase activity compared with the control (Fig. 6C, 12-48 h).

### Effect of MRW and EBL on the activities of XTH and POD during adventitious rooting

**Fig. 7:**
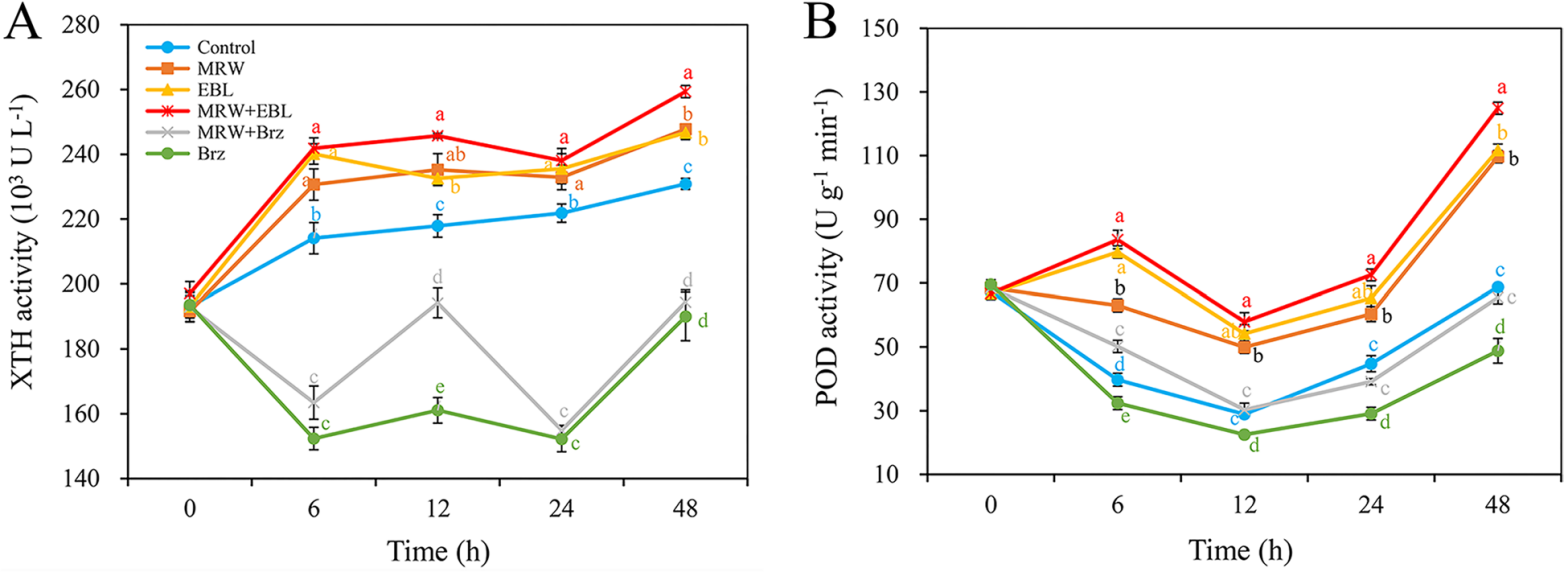
Effect of exogenous 2,4-epibrassinolide (EBL), methane-rich water (MRW) and brassinazole (Brz) on the XTH and POD activity during AR development in marigold explants. The primary roots of 5-day-old seedlings were removed, and then the marigold explants were incubated with 1 μmol L^− 1^ EBL, 20% MRW, 0.5 μmol L^− 1^ Brz (a synthetic inhibitor of EBL) alone or together for 48 h, as indicated. The activities of xyloglucan endotransglucosylase/hydrolase (XTH, A), peroxidase (POD, B) were measured at 0, 6, 12, 24 and 48 h after treatment. Values (mean ± SE) are the means of three independent experiments (*n* = 200). Bars with different lowercase letters are significantly different (Duncan’s multiple range test; *p* < 0.05)

As shown in Fig. 7A, the XTH activity in the control, MRW, EBL, and MRW + EBL treatments showed an upward trend during rooting. Compared with the control, the XTH activity in MRW, EBL, and MRW+ EBL treatment was increased by 7.7, 12.1, and 13.0% at 6 h, and was increased by 7.3, 6.8, and 12.3% at 48 h. However, Brz treatment resulted in the lowest XTH activity in all treatments between 6 and 48 h (Fig. 7A).

As shown in Fig. 7B, from 0 to 6 h, the POD activity in EBL and MRW + EBL treatments was increased, but it was decreased in other treatments. In all treatments, the POD activity steadily decreased from 6 to 12 h, and sharply increased from 12 to 48 h (Fig. 7B). The POD activity in MRW + EBL-, MRW-, or EBL-treated explants was significantly higher than that in the control explants. Compared with the control, MRW, EBL, and MRW+ EBL treatments significantly increased the POD activity from 6 to 48 h (Fig. 7B). Among all treatments, the minimum POD activity was obtained in Brz-treated explants between 6 and 48 h.

## Discussion

CH_4_ is considered as a gasotransmitter candidate similar to nitric oxide (NO), carbon monoxide (CO), and hydrogen sulfide (H_2_S), which is involved in plant development and physiological processes [27]. Our previous study has shown that MRW induced bulb formation in lily bulbs by modulating phytohormones [28]. In addition, exogenous CH_4_ promoted AR development in cucumber (*Cucumis sativus* L.), soybean [*Glycine max* (L.) Merrill], and mung bean [*Vigna radiata* (L.) Wilczek] [29] suggesting that MRW may play a key role in AR formation in plants. In this study, we demonstrated that MRW significantly increased AR number in marigold (Fig. 1C, D). The results were consistent with Cui et al. [29], who showed that MRW triggered ARs by modulating heme oxygenase 1/ carbon monoxide and calcium pathways in cucumber explants. In their experimental system, the most effective concentrations of MRW in cucumber, soybean and mung bean were 80, 50 and 10%, respectively; whereas in the present study, the most effective concentration of MRW in marigold was 20%. These results suggest that CH_4_ may induce AR formation in a plant species-dependent manner. BRs have been shown to regulate root meristem size by promoting cell cycle processes [30]. BRs promoted pollen germination in vitro and pollen tube growth rates [31]. In the present study, EBL treatment promoted AR formation in marigold explants in a dose-dependent manner, with 1 μmol L^− 1^ EBL being the most effective concentration (Fig. 1A, B), suggesting that exogenous BR may play a positive role in AR formation. This result was consistent with that of Li et al. [20], who showed that BR promoted AR formation by inducing endogenous NO production in cucumbers. In this study, brassinazole (Brz, a synthetic inhibitor of EBL) was used to investigate whether BR is involved in CH_4_-induced AR formation. The results showed that Brz inhibited the promotion of CH_4_ on AR formation (Fig. 2), indicating that BR might play an important role in CH_4_-induced AR formation in marigolds. Our previous study has shown that HRW induces the formation of lily bulbs and AR by increasing starch and sucrose synthesis [32]. BR increased NO production by regulating the activity of NR and NOS-like enzymes, thereby promoting the formation of AR in cucumber [20]. In addition, CH_4_ participated in the formation of small bulbs by regulating 3-indole acetic acid (IAA), jasmonic acid (JA), zeatin (ZT), gibberellin (GA), and abscisic acid (ABA) levels and their signal transduction [28]. In the study, the involvement of BR in CH_4_-induced AR formation is reported for the first time.

BR is one of the earliest brassinosteroids discovered and plays an important role in cell division and expansion, stem elongation, root growth, new shoot growth, and xylem differentiation [33]. Epibrassinolide (EBL), as one of the most active brassinolides, plays multiple roles in plant growth and development and biochemical pathways [34]. BR signals are sensed by the receptor BRI1/BAK1 on the cell membrane and then regulate the expression of downstream genes in response to a series of proteins and enzymes, further regulating plant growth and development [35]. In *Arabidopsis thaliana* (L.) Heynh, brassinolide is the predominant form of BR. Interestingly, we found that MRW treatment increased the endogenous EBL content in marigold explants (Fig. 3D). The synthesis pathway of BR is divided into the early C-6 oxidation pathway and the late C-6 oxidation pathway in the CN-dependent pathway. In the early C-6 oxidation pathway, campesterol (CR) is used as a synthetic precursor to produce Campestanol (CN) through a dehydrogenation reaction, followed by the intermediate substance Cathasterone (CT), and finally by Castasterone (CS) to produce Brassinolide (BR). In this study, MRW treatment significantly increased the content of CN, CT, and CS in the BR synthesis pathway (Fig. 3A, B, C), indicating that CH_4_ could induce the production of endogenous BR. He et al. showed that DWF4, DET2, and BR6ox1/2 are required for the production of BRs in the biosynthetic pathway of BR [36]. OsBR6ox, which is involved in brassinolide biosynthesis, could positively regulate AR formation in rice [37]. In this study, MRW significantly up-regulated the enzyme activity of DET2, DWF4, and BR6ox in marigold explants (Fig. 4). Therefore, we concluded that CH_4_ could promote endogenous BR production.

Cell wall is the unique cell structure of plant cells, which regulates the direction and rate of cell growth and affects the differentiation and function of various cells. Prior to maturation, plant cells usually pass through an irreversible process of cell wall expansion. The central aspect of expanded plant cell growth is the hydrolysis of the polysaccharides that make up the cell wall by a series of hydrolytic enzymes (cell wall relaxing factors), which results in cell wall relaxation. Our previous study confirmed that salicylic acid (SA) treatment increased pectin content and reduced the degree of pectin methylation under C_d_ stress, thereby promoting AR formation in cucumber [38]. Cellulose is the main load-bearing component of the primary cell wall. It is composed of long chains of β-1,4-linked glucan chains. Cellulose is produced at the plasma membrane by the cellulose synthase complex (CSC). The cesA6 mutant may only function during cell expansion [39]. Extra regulation of CESA by post-translational modifications (e.g. phosphorylation) may modulate the stability or motility of CSCs [40]. It has been shown that the protein kinase BRASSINOSTEROID INSENSITIVE2 phosphorylates CESA 1, highlighting an example of hormonal regulation of cellulose synthesis by phosphorylation of CESA [41]. In vitro experiments showed that the network formed by cellulose and xyloglucan plays an important role in the ductility and strength of incipient cell walls [42]. Xyloglucan is one of the main components of hemicellulose and is widely found in the cell walls of tissues at various stages of development, such as the primary cell wall of growing cells and secondary cell wall of differentiating cells, indicating that it plays an important role in cell wall construction [43]. In the present study, both MRW and EBL significantly reduced cellulose, hemicellulose, and lignin content at the initiation period (12 h) of AR induction. However, this down-regulation was reversed by Brz (Fig. 5). The results confirmed that CH_4_ and BR could regulate AR formation by reducing cellulose, hemicellulose, and lignin synthesis during the initiation phase. Xyloglucan has been found to play a key role in contributing to the physical properties of cell walls during plant growth [44]. Increased ethylene biosynthesis in ARs is accompanied by the activation of xylanase [45]. A number of cell wall hydrolases, namely cellulase or β-(1,4)-glucanase, pectinesterase (PG), α-arabinosidase, α- and β –galactosidase, and α-mannosidase, were detected during fruit ripening, and the activity of the enzymes increased with fruit ripening [46]. In this study, we found that MRW and EBL significantly up-regulated the activities of carboxymethyl cellulase and xylanase at the AR initiation stage (12 h), while MRW + EBL treatment significantly upregulated the activities of xylanase. Meanwhile, the positive effects of MRW and EBL were reversed by Brz (Fig. 6A, B). Lignin, which exists in the secondary wall, is secreted into the cell wall and modified by POD and laccase in the wall. In our study, the laccase activity in all treatments was up-regulated during the initiation stage (6-12 h) of AR, except that Brz treatment decreased it. The laccase activity in MRW + EBL treatment was higher than that in other treatments at 12 h (Fig. 6C). This is consistent with the results of Zhao and Kwan [47], who reported that a laccase from the edible mushroom *Lentinula edodes* plays a role in fungal morphogenesis. In addition, a laccase was specifically expressed in the green conidia of *Aspergillus nidulans* [48]. Our study also found that MRW and EBL treatments up-regulated CMCase, xylanase and laccase activity during AR expression period (24-48 h), while this up-regulation was inhibited by Brz (Fig. 6). These results indicated that CH_4_ and BR may regulate AR formation by up-regulating CMCase, xylanase, and laccase activity.

Xyloglucan endotransglycosylase/hydrolase (XTH) exhibits the activity of xyloglucan endotransglycosylase, which has been considered as a major cell wall relaxation factor [49]. Many studies have explored the function of XTH and found that it regulates fruit softening by altering the fruit cell wall. For example, overexpression of SLXTH1 reduced the firmness of tomato fruit, possibly through the depolymerization of total sugar and xylan [50]. FvXTH9 and FvXTH6 in strawberry (*Fragaria × ananassa* Duch.) are necessary to alter the xylan structure in the fruit cell wall, and the transient expression of FvXTH9 and FvXTH6 accelerated the softening process of strawberry [51]. In our study, MRW and BR treatments significantly increased XTH activity, while Brz treatment significantly decreased XTH activity (Fig. 7A). The results were consistent with those of Xu et al. [52], who reported that the *TCH4* gene which encodes xyloglucan endotransglucosylase affected cell wall formation and degradation in plant morphogenesis. POD plays an important role in cell wall structure and plasticity [53]. Our previous study found that NO or H_2_O_2_ treatment enhanced POD activity in chrysanthemum cuttings during AR initiation [19]. Sodium hydrosulfide (NaHS) treatment increased the activities of antioxidant enzymes (POD, superoxide dismutase, ascorbate peroxidase and catalase) and increased the contents of ascorbic acid and glutathione during rooting [54]. In this study, MRW, EBL and MRW + EBL treatments significantly enhanced POD activity during both the initiation phase (6-12 h) and expression phase (12-48 h), while Brz treatment significantly inhibited the positive effect (Fig. 7B). Therefore, BR may be involved in CH_4_-regulated cell wall relaxation during rooting, which may be achieved by increasing XTH and POD activity.

## Conclusions

**Fig. 8:**
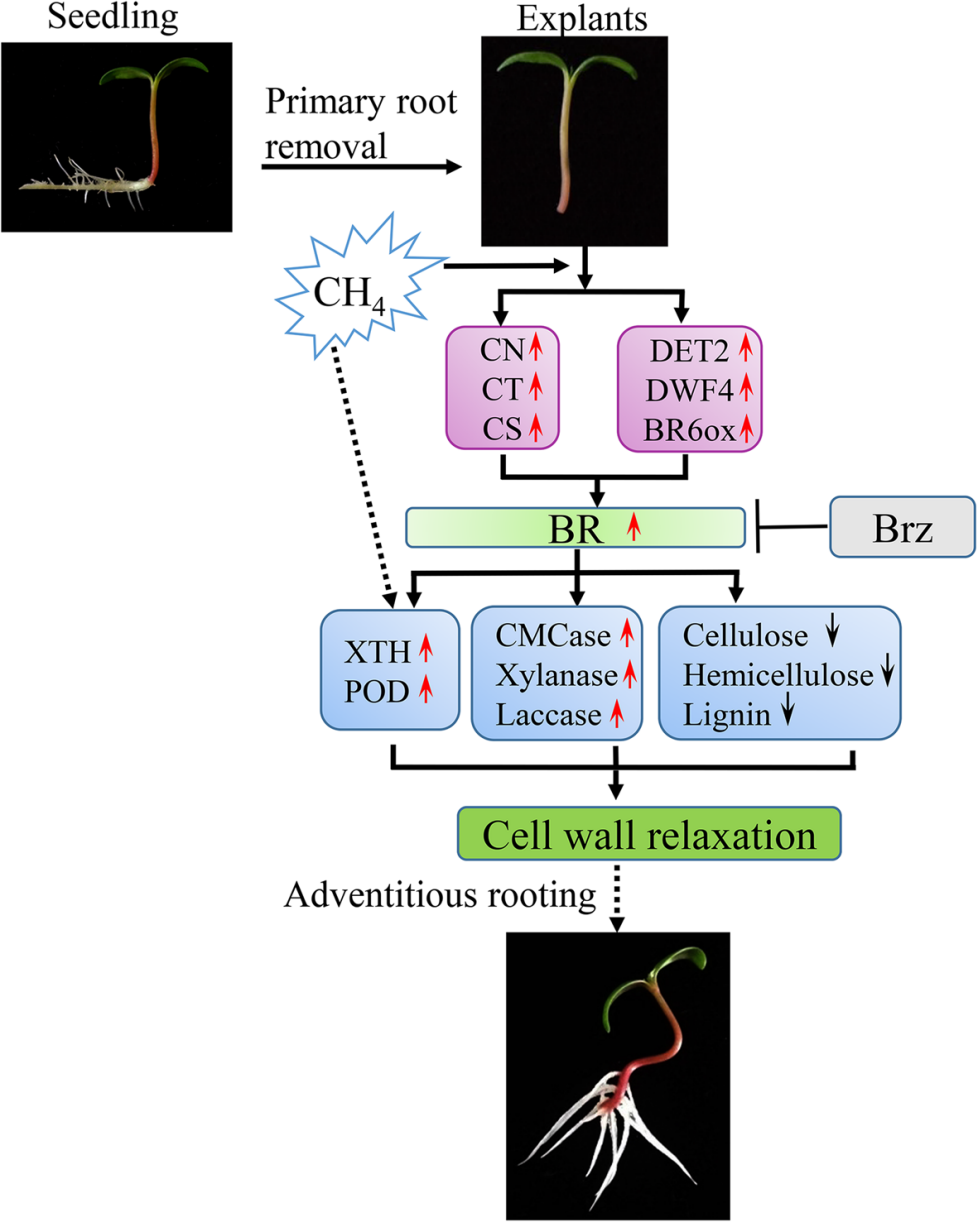
A possible mechanism for modulation of AR formation by CH_4_-triggered BR-dependent cell wall relaxation. The involvement was suggested by solid lines, and the possibility was suggested by dashed lines. T bars, inhibition

Exogenous MRW or EBL significantly promoted AR formation in marigold, and BR may play an important role in CH_4_-induced AR formation. CH_4_ could increase the content of endogenous BR by regulating the content of intermediates products and the activity of major enzymes in BR biosynthesis pathway. In addition, CH_4_ significantly regulated the content of the main cell wall components and the corresponding hydrolase activity, as well as the antioxidant enzyme activity during AR formation (Fig. 8). Meanwhile, BR might be involved in the process. Our study provided new insight into the physiological mechanisms by which BR is involved in CH_4_-induced AR formation, i.e. CH_4_ promotes AR formation by regulating cell wall relaxation through BR synthesis. Future experiments will be needed to investigate the molecular mechanisms.

## Methods

### Plant material

Marigold seeds (*T. erecta* L. ‘Marvel’) were purchased from the New Century Garden Co., Ltd., Lanzhou, China. Seeds were selected of similar size and without physical damage, and treated according to the method described before [55]. Seeds were sterilized in 5% sodium hypochlorite solution for 10 min before five washes in sterile distilled water, then were soaked in distilled water for 6 h. Finally, the seeds were transferred to moisture filter papers in Petri dishes (15 cm diameter and 2.5 cm deep); and germinated in an incubator which was maintained at 25 ± 1 °C for 5 days with a 14 h photoperiod (photosynthetically active radiation 200 μ mol s^− 1^ m^− 2^). The 5-day-old marigold seedlings with primary roots removed were used as explants and maintained under the same photoperiod and temperature conditions for another 5 days in the presence of different media indicated below. In order to keep the solution fresh, these media were changed every day. Analytical grade chemicals used in the research were sourced from Chinese companies. The root number and length (>1 mm) per explant were counted and measured.

### The preparation of methane-rich water solution

According to the previous method [28], methane-rich water (MRW) was prepared from 99.999% pure CH_4_ gas, which was released from a compressed gas cylinder, was bubbled into 500 ml distilled water for at least 30 min, to obtain the saturated stock solution (100% MRW). Afterward, the saturated stock solution was immediately diluted to 5, 10, 20, 50, and 100% (v/v) saturation.

### Explant treatments

The explants were treated with various concentrations of methane-rich water (MRW 0, 10, 20, 50, and 100% [v/v]), 2,4-epibrassinolide (EBL; 0, 0.05, 0.1, 0.5, 1, 5 μmol L^− 1^; Solarbio, Beijing, China), brassinazole (Brz, a synthetic inhibitor of EBL; 0, 0.05, 0.1, 0.5, 1, 5 μmol L^− 1^; Sigma, United States), respectively. The experiment treatment is as follows: the control (distilled water), 20% MRW, 1 μmol L^− 1^ EBL, 20% MRW + 1 μmol L^− 1^ EBL, 20% MRW + 0.5 μmol L^− 1^ Brz, and 0.5 μmol L^− 1^ Brz. Three repetitions of each treatment are performed. The concentrations of these chemicals were selected based on preliminary experimental results. Finally, marigold explants were taken at 0, 6, 12, 24, and 48 h of the incubation for subsequent assays.

### Measurement of endogenous EBL, CN, CT, and CS content

The extract solution was performed with slight modifications on the basis of the previous method [28, 56]. Briefly, fresh explants (0.5 g) were ground to a fine powder and extracted in 5 mL ice-cold 80% (v/v) methanol at 4 °C avoiding light for 12 h. After centrifugation at 4000 *g* for 15 min at 4 °C, the supernatant was filtered and the residue was re-extracted twice with another 5 mL of pre-chilled methanol, then pooled all supernatants. The supernatant (2 mL) was evaporated under a vacuum concentration at 4000 *g* for 4 h at 38 °C. The dry extract was redissolved in 1 mL of 80% methanol, and the dissolved solution was filtered by a 0.22 μm microporous membrane, the extract solution was collected for further analysis. Endogenous EBL was quantified by liquid chromatography-mass spectrometry equipped with a C-18 column (2.1 × 50 mm, 1.8 μm, Agilent 1290 Series). The mobile phase consisted of 0.1% formic acid and chromatographic acetonitrile at a flow rate of 0.3 ml min^− 1^. The column temperature and injection volumes were 30 °C and 2.0 μL, respectively.

According to the manufacturer’s instructions, campestanol (CN), cathasterone (CT), and castasterone (CS) content was measured by enzyme-linked immunosorbent assay (ELISA; Cheng Lin Biological Technology Co. Ltd., Beijing, China).

### Measurement of cellulose, hemicellulose and lignin content

The quantification of cellulose content was conducted by a modified titration method [57]. Briefly, an air-dried sample equivalent to 0.5-1.0 g was weighed, 5 mL of a mixture of nitric acid and glacial acetic acid was added, and the reaction mixture was stirred periodically in boiling water for 25 min and cooled to room temperature. To the precipitate, 10 mL of 10% sulfuric acid in 0.1 mol L^− 1^ potassium dichromate solution was added, and the mixture was immersed in boiling water for 10 min, removed and poured into a conical flask. Three drops of o-phenanthroline reagent were added to the reaction mixtures, and the solution was titrated with 0.1 N ammonium ferrous sulfate. The cellulose content, expressed as %, was calculated using formula (1).

1 $$\textbf{Cellulose}\ \textbf{content}\ \left(\%\right)=\textbf{0.00675}\times \textbf{k}\times \left(\textbf{a}-\textbf{b}\right)/\textbf{n}\times \textbf{100}\%$$

Where 0.00675 is the standard titration of cellulose (1 mL of standard potassium dichromate equivalent to 0.00675 g of cellulose [57]); K is the titration of ferrous ammonium sulfate (the titration of ferrous ammonium sulfate is determined on the day of use); a is the amount of ferrous ammonium sulfate consumed in the blank (ml); b is the amount of ferrous ammonium sulfate consumed in the sample (ml); n is the weight of the air-dried sample (g).

The quantification of hemicellulose content was determined by employing the method of Van Soest [58] with slight modification. Fifteen mL 80% calcium nitrate solution was add to 0.1-0.2 g of the air-dried sample powder. The reaction mixture was heated in water to micro-boiling for 5 min. Then, 10 mL 2 N hydrochloric acid was added to the precipitate, and the mixed solution was invaded into the boiling water for 45 min. After cooling to room temperature, it was centrifuged and the supernatant was combined with 10 mL distilled water. The hemicellulose content was obtained according to formula (2).

2 $$\textbf{Hemicellulose}\;\textbf{content}\;\left(\%\right)=\textbf{0.009}\times\textbf{100}\times\left[\textbf{248}-\left(\textbf{a}-\textbf{b}\right)\right]\times\left(\textbf{a}-\textbf{b}\right)/\mathbf{10}^{\mathbf5}\times\textbf{n}\times\textbf{100}\%$$

Where 0.009 is the standard titration of hemicellulose; a is the amount of sodium thiosulfate consumed in the blank (mL); b is the amount of sodium thiosulfate consumed in the sample (mL); n is the weight of the air-dried sample (g).

The lignin content was determined according to Kratzl and Billek’s method [59] with slight modification. The air-dried sample powder (0.05-0.1 g) was put into a centrifuge tube, and 10 mL of 1% acetic acid was added. The mixture was shaken for 5 min and centrifuged. The precipitate was washed with 5 mL 1% glacial acetic acid and centrifuged. The lignin content was expressed as % and calculated by formula (3).

3 $$\textbf{Lignin}\ \textbf{content}\ \left(\%\right)=\textbf{0.00433}\times \textbf{K}\times \left(\textbf{a}-\textbf{b}\right)/\textbf{n}\times \textbf{100}\%$$

Where 0.00433 is the standard titration of lignin; and k, a, b, and n are the same as described in the above method of the determination of cellulose content.

### Enzyme activity assay

#### Steroid 5α-reductase (DET2), 22α-hydroxylase (DWF4), and BR-6-oxidase (BR6ox) enzyme activity measurement

The enzyme solution was extracted according to Jin et al. [60] with slight modifications. Briefly, 0.5 g frozen explants were ground in 5 ml of 50 mmol L^− 1^ cool phosphate buffer (pH 7.0) in a mortar and pestle, containing 1% (w/v) polyvinylpolypyrrolidone (PVPP) and 1 mmol L^− 1^ ethylenediaminetetraacetic acid (EDTA). The homogenate was centrifuged at 4000 *g* for 15 min at 4 °C. The supernatant was gathered as the crude enzyme for the further enzymatic activity assays.

The activities of DET2, DWF4, and BR6ox were analyzed by plant DET2 assay kit, plant DWF4 assay kit, and plant BR6ox assay kit, respectively, according to the manufacturer’s instructions. The kits were purchased from Beijing Chenglin Biological Engineering Co, China. Measurements were done in 3 independent biological replicates.

#### Hydrolase activity assay

According to Chin et al. [46] with slight modifications, the extraction of enzymes was carried out at 4 °C. Fresh explants (0.5 g) were finely ground in liquid nitrogen with 5 ml of the extract mixture (0.05 mol L^− 1^ citric acid buffer, PH 5.0, 10 m mol L^− 1^ β-mercaptoethanol, and 1% (w/v) Polyvinyl Polypyrrolidone (PVP), and 30 m mol L^− 1^ sodium ascorbate). The supernatant was recovered by centrifugation (10,000 *g* for 20 min); and used to determine enzyme activity.

Cellulase (CMCase) activity was assayed by the method described by Chin, Ali, & Lazan [46]. The reaction mixture contained 0.1 ml of crude enzyme, 0.15 ml 1% (w/v) carboxymethyl cellulose, and 0.5 ml 0.1 mol L^− 1^ citric acid buffer, pH 5.0. Incubation was carried out at 50 °C for 30 min followed by the addition of DNS (3,5-dinitrosalicylate) reagent. After 5 minutes of heating in a boiling water bath, the absorbance was measured at 540 nm with glucose as a standard [61].

Assays for xylanase were performed according to the modification of the method of Bailey, Biely, and Poutanen [62]. The assay mixture consisted of 1.8 mL 0.05 mol L^− 1^ citric acid buffer containing 1% xylan substrate, pH 5.0, 0.2 mL enzyme solution, and incubated at 50 °C for 10 min. The reaction was stopped by adding 3 mL of DNS reagent boiling water for 10 min, and the amount of xylose formed was determined from the absorption at 540 nm. Specific activity is expressed in units per gram of fresh plant. One unit is the amount of enzyme required to release 1 μmol of xylose per minute.

The laccase activity was determined by monitoring the increase in absorbance at 420 nm as described by Bhattacharya and Banerjee with slight modifications [63]. The analytical mixture contained 1 mmol L^− 1^ 2,2′-azinobis (3-ethylbenzothiazoline-6-sulfonic acid ammonium salt, ABTS) in 0.1 mol L^− 1^ sodium acetate buffer (pH 4.5) and 0.1 mL of enzyme samples. One unit of enzymatic activity (U) was defined as the amount of enzyme required to oxidize 1 μmol L^− 1^ of ABTS (ε 420 = 36,000 mol ^− 1^ cm ^− 1^) per minute under the analytical conditions.

The activity of xyloglucan endotransglucosylase/hydrolase (XTH) was analyzed following the method described by a plant XTH assay kit (Cheng Lin Biological Technology Co. Ltd., Beijing, China). Measurements were done in 3 independent biological replicates.

#### Peroxidase activity determination

The peroxidase (POD) activity was determined using a modified procedure of Han et al. [64]. Fresh explants (0.5 g) were ground in a mortar and pestle in 5 mL of 0.5 mol L^− 1^ cool phosphate buffer (pH 7.0), containing 1% (w/v) polyvinyl polypyrrolidone (PVP) and 1 mmol L^− 1^ ethylenediaminetetraacetic acid (EDTA). The homogenates were centrifuged at 10000 g for 20 min at 4 °C, and the supernatants collected was crude enzyme fluid. The reaction mixtures containing 2 mL guaiacol substrate (8 mmol L^− 1^ guaiacol and 0.1 mol L^− 1^ phosphate buffer, pH 7.0), 1 mL 24 mmol L^− 1^ H_2_O_2_, and 0.5 ml crude extract. The buffer solution was used as a control instead of the enzyme solution, and the rate of absorbance was measured at 470 nm. Finally, one unit of the activity of the POD enzyme was defined as the change in the absorbance in 0.01 units per min, and POD enzyme activity was denoted as U g^− 1^ min^− 1^.

### Statistical analysis

A completely randomized experimental design was used in the study. All of data presented in the figures were expressed as the mean values ± SE from three independent biological replicates. Data collected were subjected to analysis of variance (ANOVA), and statistical divergence among treatments was analyzed through Duncan’s multiple range test. All statistical analysis was carried out using the statistical package for social science for windows (version 22.00; SPSS, Inc., Chicago, IC, United States).

## Data Availability

The data that support the findings of this study are available from the corresponding author on reasonable request.

## Abbreviations

CH_4_: Methane
BRs: Brassinosteroids
BR: Brassinolide
AR: Adventitious root
MRW: Methane-rich water
EBL: 2,4-epibrassinolide
Brz: Brassinoazole
CR: Campesterol
CN: Campestanol
CT: Cathasterone
CS: Castasterone
DET2: 5α-reductase
DWF4: 22α-hydroxylase
BR6ox: BR-6-oxidase
CMCase: Carboxymethyl cellulose
XTH: Endotransglucosylase/hydrolase
POD: Peroxidase
HRW: Hydrogen-rich water
NO: Nitric oxide
NR: Nitrate reductase
NOS: NO synthase
CO: Carbon monoxide
H_2_O_2_: Hydrogen peroxide
